# An adaptation of a macroscale methodology to assess the direct economic losses caused by Tropical Cyclone Idai in Zimbabwe

**DOI:** 10.4102/jamba.v14i1.1276

**Published:** 2022-09-26

**Authors:** Emmanuel Mavhura, Komal R. Aryal

**Affiliations:** 1 Department of Geography, Faculty of Science and Engineering, Bindura University of Science Education, Bindura, Zimbabwe; 2 Research and Innovation Centre, Faculty of Resilience, Rabdan Academy, Abu Dhabi, United Arab Emirates

**Keywords:** Tropical Cyclone Idai, direct economic loss, estimate, sector, Zimbabwe

## Abstract

Tropical cyclones are among the costliest disasters in the world, with reported losses amounting to billions of US dollars on an annual basis. To reduce the impact of disasters including cyclones, Zimbabwe signed the Sendai Framework whose Target C is aimed at reducing the direct economic losses of disasters. Under the direction of the United Nations Office for Disaster Risk Reduction (UNDRR), an open-ended intergovernmental expert working group (OIEWG) developed a simple methodology for estimating direct disaster-economic loss. Therefore, this study tested the applicability of the OIEWG methodology in assessing the direct economic losses induced by Tropical Cyclone Idai (TCI) in Zimbabwe. The results revealed that TCI inflicted huge losses in most sectors of the economy, notably the housing, agriculture and the critical infrastructure. The sectoral analysis approach of the OIEWG methodology worked well in distinguishing direct and indirect loses as well as in underlining the need to adopt and effectively implement adequate risk reduction strategies in the built environment. Strengthening such strategies such as the ‘build back better’ principle, cyclone forecasting and warning systems and constructing cyclone-resilient infrastructure is critical in order to minimise direct losses attributed to cyclones.

## Introduction

Worldwide, tropical cyclones (TCs) have dominated weather-related disaster damages during the past two decades (Hudson et al. 2019). The TCs develop in tropical and subtropical regions where they are referred to as hurricanes, typhoons or willy-willies (Van der Sommen, Pearson & Boggs 2018). On a yearly basis, these storms create billions of dollars in damages across many places (Chari, Ngcamu & Novukela 2020; Ishizawa, Miranda & Strobl 2019). In Southern Africa, TCs are the second-most prevalent and impactful disaster, after drought (Centre for Research on the Epidemiology of Disasters [CRED] 2019). Between 1998 and 2017, the TC became the costliest disaster, with reported losses amounting to US$1.3 billion in Southern Africa (Wallemacq, House & McClean 2018). This amounted to 46% of recorded economic losses of all hydrometeorological and geologic disasters during that period. The economic losses induced by TC include damage to or destruction of infrastructure, family assets and ecosystems (Hoque et al. 2017). They also interrupt individual or family consumption behaviour, government expenditures and investment decisions (Haque & Jahan 2016). The damages are a combined action of high destructive winds, torrential rains, landslides, storm surges and flooding (Hoque et al. 2016).

The impact of TC is a function of several factors, including the physical characteristics of the storm itself, economic characteristics of the affected place, its topography and the preparedness level of the affected community, amongst others (Bueti, Ginis & Rothstein 2014; Duvat, Volto & Salmon 2017; Kabir, Salehin & Kibria 2015). As evidenced in Seychelles, high magnitude TC (Category V storms according to the Saffir-Simpson scale) can generate strong surface winds and heavy rains that can cause huge economic damages (Duvat et al. 2017). Economically developed regions tend to suffer greater losses than the less-developed ones (Haque & Jahan 2016; Ishizawa et al. 2019). Furthermore, economies with many interindustry linkages can suffer severe disruptions which can spread to neighbouring countries (Van der Sommen et al. 2018).

In March 2019, Cyclone Idai, a category IV storm, landed in Zimbabwe through Mozambique (Mavhura 2020). It was the worst storm to hit Zimbabwe in the last five decades. The cyclone brought very strong winds of about 195 km/h (120 mph), torrential rains and floods, which caused extensive damage to public utilities, residential areas and agriculture (Munsaka et al. 2021). The water, sanitation and hygiene (WASH) infrastructure in individual homes, health and educational institutions and public buildings were extensively damaged. Amongst the damaged infrastructures, the most critical were water pumping stations and reticulation networks (World Bank 2020). The cyclone also induced landslides and mudslides, which cut off the roads and buried more than 70% of houses in the townships of Chimanimani and Chipinge districts (Chanza et al. 2020). For close to a month, some communities in Chimanimani district were isolated because of blocked roads, a situation that forced the government of Zimbabwe to airlift relief materials using the army. Other families went for more than a year living in temporary shelters without electricity, running water and other basic services (Dube, Wedawatta & Ginige 2021). To date, a couple of studies on Cyclone Idai have been carried out, focusing on what went wrong and right during the disaster in Zimbabwe (Chanza et al. 2020; Dube et al. 2021; Mavhura 2020; Munsaka et al. 2021); however, the direct economic losses of the storm have not been ascertained in order to inform disaster risk reduction (DRR) policy and practice in Zimbabwe.

Under the direction of the United Nations Office for Disaster Risk Reduction (UNDRR), an open-ended intergovernmental expert working group (OIEWG) developed methodological guidelines for determining direct disaster economic loss for Target C of the Sendai Framework for Disaster Risk Reduction (SFDRR) (UNDRR 2017). Although the OIEWG methodology was principally designed to assist countries in consistently monitoring their progress in achieving Target C of the SFDRR (UNDRR 2017), it can be adapted at a microscale to estimate the disaster economic losses and therefore inform DRR policy and practice. Furthermore, the methodology has not been widely tested in African countries. Therefore, this study explicitly tested the applicability of the OIEWG methodology by assessing the direct economic losses induced by Tropical Cyclone Idai (TCI) in Zimbabwe. Thus, the key questions driving the study were: in what ways did TCI directly affect the economy of Zimbabwe? How can the OIEWG methodology for Target C be applied to determine the direct economic losses induced by TCI in Zimbabwe? The next section will discuss some perspectives and challenges in determining economic losses from disasters.

## Determining disaster economic losses

Economic losses from disasters are either direct or indirect. Direct losses involve the ‘physical damages and destruction of physical assets’ while indirect losses are ‘interruptions of supply chain as a result of disasters’ (Okuyama 2007:116). Thus, direct economic losses are derived from tangible assets such as roads, bridges and crops, while the indirect losses are intangible and include loss of income or revenue to business interruption or missing assets (UNDRR 2017).

In many cases, the determination of economic losses in the aftermath of a disaster is performed using post disaster needs assessment (PDNA) methodologies. Depending on the scale of the disaster, two PDNAs may be conducted, a rapid needs assessment and a more comprehensive one. The rapid needs assessment is usually completed within 72 days of the disaster taking place, while a more comprehensive PDNA takes several weeks or months. The rapid assessment may not be accurate because it is usually hurriedly performed in order to provide statistics for relief, humanitarian appeals and solidarity aid (Longenecker et al. 2020; Yuan & Liu 2018). A more comprehensive PDNA provides detailed and more reliable economic loss data; however, many of the comprehensive PDNA are only calculated after large-scale disasters. Yet a significant number of small-scale and recurring events that negatively impact communities rarely receive such comprehensive assessments and documentation (Osuteye, Johnson & Brown 2017). Some under-estimations and over-estimations of the actual loss are likely to occur during PDNA (Natho & Thieken 2018). In addition, defining the timeframe at which reconstruction costs of the damaged or destroyed physical assets is attributed to the disaster(s) is a challenge.

There are many country-specific and sector-specific methodologies of estimating economic losses induced by disasters. Firstly, Ishizawa et al. (2019) used monthly night light images to calculate the economic impact of TC in the Dominican Republic. Although this method is effectively used where there is limited public data, it has one major limitation: it is mainly restricted to yearly data, partly because such night light data are usually available on yearly basis. This tends to mask most of the economic damages within the year under study (or at other higher temporal frequencies). Secondly, Haque and Jahan (2016) used an input–output economic modelling framework to estimate losses from Cyclone Sidr in Bangladesh. Although the model managed to comprehensively trace the interactions amongst various sectors of the Bangladesh economy using relatively less data, it had two major weaknesses. Firstly, the model maintained a linear and static nature and secondly, it failed to respond to changing prices of goods and services and the economies of scale experienced during production. Other technical models that are used to determine disaster economic losses include the computable general equilibrium (CGE) and the social accounting matrix (SAM) (Mainar-Causapé, Ferrari & McDonald 2018; Xie et al. 2014b). Although the CGE techniques are nonlinear and capable of factoring in changes in prices of goods and service, they are well-suited for long-run equilibrium analyses, a situation which often leads to underestimation of the disaster losses. Unlike the CGE technique, the SAM model is too rigid and requires a large amount of data in order to come up with upper bounds for the estimates (Okuyama 2007). Furthermore, both the SAM and CGE techniques hardly distinguish between direct and indirect losses.

In addition, some countries use replacement costs, that is, the cost of repairing or replacing the damaged buildings or assets with materials of almost the same kind and quality, to estimate the disaster loss (Natho & Thieken 2018). Although the replacement method might be easy to calculate, it may provide values that are more than the actual physical loss because of price increases soon after a disaster. Other countries, especially in Europe, use insurance compensation mechanisms to determine direct economic losses of a disaster in the housing sector (Surminski & Thieken 2017). However, asset insurance is rarely available or is not affordable in many developing countries, including Zimbabwe. In Europe, countries such as Germany use cereal yield and price per ton data to calculate losses in the agriculture sector (Natho & Thieken 2018). As for the roads sector, Germany uses the length of the affected paved roads as a variable to calculate direct economic losses. In addition to these methods, some advanced countries use remote sensing techniques to estimate the direct disaster-economic losses (Fan et al. 2017).

In view of the given perspectives and challenges, determining the actual economic loss is a complicated matter. While direct damage and destruction to infrastructure and assets are relatively easy to monetise (i.e. conversion of physical value into economic value), intangible costs such as loss of biodiversity, stress and inconveniences caused are difficult to measure in monetary terms (Hudson et al. 2019). Furthermore, some disasters create non-linear cascading effects over time that are hard to capture during the PDNA. For example, the Chinese Ice Storm of 2008 resulted in significant losses in the demand and supply sides of industry and commerce, which were difficult to measure when the disaster occurred (Xie et al. 2014a). In other cases, disasters bring in economic benefits through donations, remittances, relief funds and humanitarian assistance that may outweigh the costs incurred by the affected regions. For example, following the 2004 Indian Ocean Tsunami disaster, the international humanitarian response brought in untied aid of about US$13.5 bn, which exceeded the estimated losses and damages of about US$10 bn (Telford & Cosgrave 2007). Likewise, flooding in the Muzarabani district of Zimbabwe creates residual moisture, which brings many economic benefits to riparian communities who depend on off-rainy season farming (Mavhura 2017). Therefore, in such situations where disasters bring in certain benefits to the affected communities, there may not be any incentive to undertake economic loss estimates induced by the disasters. Furthermore, many developing countries do not have the capacities to consistently quantify the economic loss (Natho & Thieken 2018).

## Methodology

### Data source

This study relied on secondary data from reports published by the government (Department of Civil Protection [DCP] 2019), United Nations (UN) agencies (United Nations Office of the Coordination of Humanitarian Affairs [UN OCHA] 2020a, 2020b; United Nations Development Programme [UNDP] 2019), the World Bank Group (2019, 2020) and non-governmental organisations (NGOs) operating in Zimbabwe (Chatiza 2019; Tsuro Trust 2020). It strictly used reports, which followed detailed assessment methodologies such as the PDNA and the damage, loss and need assessment, which are robust and globally accepted (Deen 2015; Jeggle & Boggero 2018). The assessment focused on the economic losses (damages and or destruction) induced by TCI on the following key sectors: agriculture, services, housing, critical infrastructure and cultural heritage. By damage this study refers to minor damages to structures (not structural or architectural), which may require repair and cleaning, while destruction refers to structures that were ‘knocked down, buried, washed away’ (UNDRR 2017:46). In cases where there were differences in statistics amongst the data sources, this study used government figures.

### Data analysis

This study adapted the OIEWG methodology for Target C at a micro level in order to determine the direct economic loss attributed to TCI in Zimbabwe (C_1_). The key principle behind the adapted OIEWG methodology is to convert the physical damage or destruction to units, assets or sectors of the economy into monetary value using the replacement costs (UNDRR 2017). Therefore, the study first disaggregated the economic losses into agriculture (C_2_), service sectors (C_3_), housing (C_4_), critical infrastructure (C_5_) and cultural heritage (C_6_). In this way, the numbering of the indicators was maintained (C_1_: compound indicator) through the disaggregated indicators (C_2_ to C_6_) as proposed by the OIEWG. Table 1 shows the adapted indicators and the additional disaggregation used for more precise estimation of losses.

**TABLE 1:** Adapted indicators of direct economic loss attributed to Tropical Cyclone Idai in Zimbabwe.

No.	Indicator	Specific disaggregation for more precise estimation of losses
C_1_	Direct economic loss attributed to TCI (compound indicator)	Agriculture, services, housing, critical infrastructure and cultural heritage
C_2_	Direct economic loss in agriculture attributed to TCI (disaggregated indicator)	Crops (maize, sorghum, pearl millet finger millet, ground nuts, round nuts, sugar beans, bananas, pineapple, mangoes, oranges and macadamia nuts); irrigation infrastructure; livestock (cattle, goats, sheep, poultry); forestry (plantations, stored timber)
C_3_	Direct economic loss in service sectors attributed to TCI (disaggregated indicator)	Educational, health and water sanitation and hygiene (WASH) facilities
C_4_	Direct economic loss in the housing sector attributed to TCI (disaggregated indicator)	All dwelling units in urban and rural areas
C_5_	Direct economic loss resulting from damaged or destroyed critical. infrastructure attributed to TCI (disaggregated indicator)	Utility infrastructures such as road transport, power and energy and telecommunication, as well as their related fixed assets
C_6_	Direct economic loss to cultural heritage damaged or destroyed attributed to TCI (disaggregated indicator)	Fixed cultural heritage assets (buildings, monuments) and moveable assets (historical artworks, artefacts)

*Source*: Adapted from United Nations Office for Disaster Risk Reduction (UNDRR), 2017, *Technical guidance for monitoring and reporting on progress in achieving the global targets of the Sendai Framework for Disaster Risk Reduction,* viewed 15 March 2021, from https://www.unisdr.org/files/54970_techguidancefdigitalhr.pdf.

TCI, Tropical Cyclone Idai.

Firstly, the disaggregated indicators were calculated, beginning with the direct agricultural loss (C_2_) caused by TCI. As shown in Table 1, the authors focused on the value of the most important perennial and seasonal crops and irrigation infrastructure, forestry and livestock, which were either damaged or destroyed by the cyclone. The seasonal crops include maize (*Zea mays L.*); traditional grains such as sorghum (*Sorghum bicor*), pearl millet (*Pennisetum typhoides, P. Americana* or *P. glaucum*), finger millet (*Eleusine coracana*); ground nuts, round nuts and sugar beans, while perennial crops include bananas, pineapple, citrus fruit trees such as mangoes, oranges and macadamia nuts. The loss in crops was calculated as a sum of the pre-TCI value of the destroyed or damaged crops, the value of the stored inputs and the repair or replacement costs of the other damaged or destroyed crop assets, excluding irrigation infrastructure. The loss in irrigation infrastructure was treated separately from other assets by establishing its repair or replacement costs. The loss in the forestry sector was determined by calculating the pre-TCI value of destroyed or damaged plantations, stored timber and the value of replacing or repairing damaged or destroyed assets which were used for timber production. As for the loss in livestock, the researchers calculated the sum of the pre-TCI value of the destroyed or damaged livestock inputs, assets and the value of the dead livestock. Equation 1 was used to compute the direct agricultural loss.


$${\text{C}}_{2}=\sum {\text{C}}_{2\text{c}},{\text{C}}_{2\text{I}},{\text{C}}_{2\text{L}},{\text{C}}_{2\text{F}}$$ [Eqn 1]

Where:

C_2_ is direct agriculture loss.

C_2C_ is direct crop loss.

C_2I_ is irrigation infrastructure.

C_2L_ is direct livestock loss.

C_2F_ is direct loss to forestry.

The direct economic loss to service sector assets damaged or destroyed by TCI was calculated. To do so, firstly, the services were disaggregated into education, health and water, sanitation and hygiene (WASH) sectors. Then, using guidelines from the Ministry of Local Government and Public Works (MLGPW), Zimbabwe, the educational, health and WASH infrastructure damaged or destroyed by the cyclone were converted into monetary value of the replacement cost. The values of educational assets such as textbooks and computers could not be determined because of lack of sufficient data. Equation 2 was used to compute the direct economic losses in the service sector (C_3_).


$${\text{C}}_{3}=\sum {\text{C}}_{3\text{E}},{\text{C}}_{3\text{H}},{\text{C}}_{3\text{W}}$$ [Eqn 2]

Where:

C_3_ is the direct economic loss in the service sector induced by TCI.

C_3E_ is the loss to educational facilities.

C_3H_ is the direct loss to health facilities.

C_3W_ is the direct loss to WASH facilities.

As a way of improving the accuracy of the determination of the direct loss in the housing sector, the physical loss was disaggregated into destroyed and damaged, structural type and rural and urban. This is because some rural dwellings in Zimbabwe are usually constructed of poor materials such as pole and dagga with thatched roofs, whereas urban housing units are built of more strengthened materials. Guided by the construction costs provided by the MLGPW, Equation 3 was used to approximate the direct economic losses in the housing sector (C_4_).


$${\text{C}}_{4}=\sum {\text{C}}_{4\text{a}},{\text{C}}_{4\text{b}}$$ [Eqn 3]

Where:

C_4_ is the direct economic loss in the housing sector attributed to TCI.

C_4a_ is the economic value of loss in houses damaged by TCI.

C_4b_ is the economic value of loss in houses destroyed by TCI.

As indicated in Table 1, critical infrastructure losses consisted of linear and non-linear elements, which were either damaged or destroyed by TCI. The linear infrastructure included roads, energy and telecommunication lines, while the nonlinear assets included transformers and mobile network boosters. Therefore, the estimation of direct critical infrastructure loss was based on the replacement or repair cost of the total length of the damaged or destroyed linear and nonlinear infrastructure. Firstly, the loss in the road network was estimated by multiplying the length of the damaged or destroyed road networks by the rehabilitation cost per unit length. Secondly, the costs of rehabilitating other nonlinear elements were added to the road network (e.g. bridges) in order to have a full cost of the road transport sector. The same thing was applied to energy and telecommunication sectors. Finally, the direct critical infrastructure loss (C_5_) caused by TCI was calculated using Equation 4.


$${\text{C}}_{5}=\sum {\text{C}}_{5\text{T}},{\text{C}}_{5\text{EP}},{\text{C}}_{5\text{TE}}$$ [Eqn 4]

Where:

C_5_ is the direct economic loss resulting from damaged or destroyed critical infrastructure attributed to TCI.

C_5T_ is the loss to the road transport infrastructure.

C_5EP_ is the loss to the energy and power infrastructure.

C_5TE_ is the loss to the telecommunication infrastructure.

Although most of the cultural heritage losses were intangible and indirect, a proxy of the associated direct economic loss was determined by first disaggregating the sector into movable (e.g. artwork and historical artefacts) and immovable assets (e.g. buildings, monuments and fixed infrastructure). Then, the cost of replacing the assets to a level similar to one before the TCI was calculated. Equation 5 was used to compute the loss.


$${\text{C}}_{6}=\sum {\text{C}}_{\text{6a}},{\text{C}}_{6\text{b}}$$ [Eqn 5]

Where:

C_6_ is the direct economic loss to cultural heritage induced by TCI.

C_6a_ is the economic value of nonmovable assets damaged or destroyed by TCI.

C_6b_ is the economic value of movable elements damaged or destroyed by TCI.

Finally, the direct economic loss attributed to TCI was calculated as a compound indicator (C_1_) using a computational methodology which involved a simple sum of disaggregated indicators C_2_ to C_6_ in relation to the gross domestic product (GDP) of Zimbabwe in 2019 (Equation 6).


$$C1=\frac{\sum \text{C}2,\text{C}3,\text{C}4,\text{C}5,\text{C}6}{GDP}$$ [Eqn 6]

Where:

C_1_ is direct economic loss attributed to TCI.

C_2_ is direct agricultural loss attributed to TCI.

C_3_ is direct economic loss in service sectors damaged or destroyed by TCI.

C_4_ is direct economic loss in the housing sector attributed to TCI.

C_5_ is direct economic loss resulting from damaged or destroyed critical infrastructure attributed to TCI.

C_6_ is direct economic loss to cultural heritage damaged or destroyed attributed to TCI.

GDP is gross domestic product.

The next section is about the presentation and analysis of the results.

### Ethical considerations

This research was approved and cleared by the Research Protocols Committee in the Department of Geography, Bindura University of Science Education (reference number: REC 012/2021).

## Results

The results of this study are first presented as per disaggregated sector of the direct economic loss attributed to TCI. The disaggregation enabled understanding the needs of the specific sectors of the economy which were hard hit and therefore needed prioritisation during the recovery and rehabilitation stages. The disaggregation also enabled designing the policy implications needed for DRR. After the disaggregation, the overall economic loss (compound indicator) is discussed next.

### Direct economic loss in agriculture (C_2_)

**FIGURE 1:**
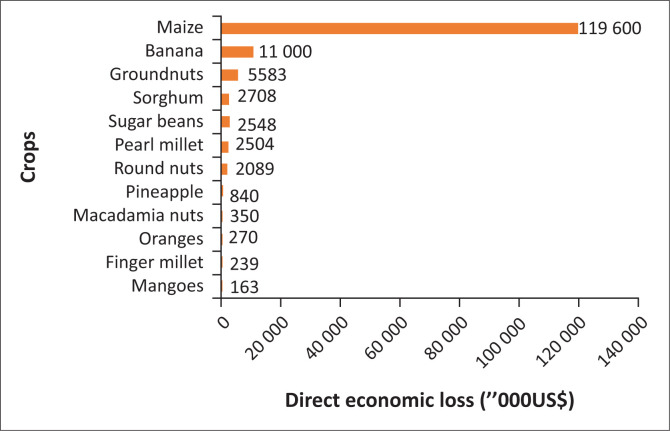
Direct economic loss in crops attributed to Tropical Cyclone Idai in Zimbabwe.

The TCI induced about US$155.4 million loss in the agriculture sector. When disaggregated by subsectors, crops accounted for about 95% of the total loss, probably because most crops (especially maize, from which the staple food in Zimbabwe is derived) were about to be harvested when the cyclone landed. The remaining 5% is shared by irrigation infrastructure (3%), livestock (1%) and forestry 1%. The irrigation infrastructure lost to TCI included the 18 irrigation schemes in Chimanimani and Chipinge districts. However, the value of farming inputs such as seeds, chemical fertilisers, feed and fodder, which were in stock before TCI, could not be ascertained; hence, they were not included in this analysis. Figure 1 shows the direct economic loss from each of the crops. The major losses came from the maize crop (88%), valued at US$119.6 m, and bananas, valued at about US$11 m.

Table 2 shows the statistics and economic value of the livestock directly lost to TCI. Most losses came from cattle, followed by poultry, sheep and goats. The aggregate loss of livestock because of TCI was valued at about US$529 135. However, these statistics should be read with caution because they are not based on numbers of bodies counted after the disaster. Rather, they are based on estimates provided by the affected farmers during the PDNA. Some farmers might have inflated the numbers in order to get compensation. In addition, the value of each livestock animal depends on its health condition, although this study used an average price to determine the total value. Hence, the actual value might be more or less what is stated here.

**TABLE 2:** Direct economic loss in livestock attributed to Tropical Cyclone Idai in Zimbabwe.

Livestock group	Number	Total loss value (USD)
Cattle	1362	426 300
Poultry (chickens, turkeys and guinea fowls)	12 413	67 215
Sheep	561	33 660
Goats	49	1960

**Total**	**14 385**	**529 135**

### Direct economic loss in service sectors (C_3_)

**FIGURE 2:**
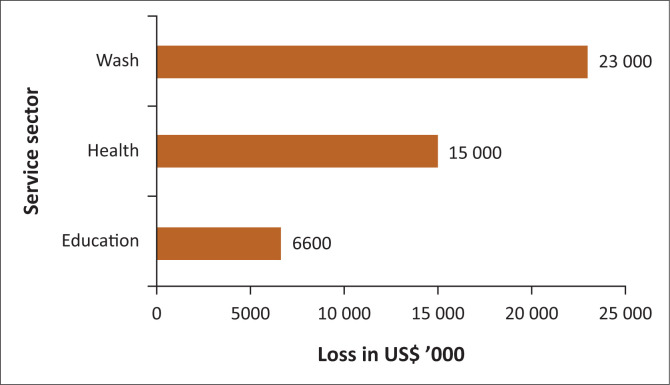
Direct economic loss in service sectors attributed to Tropical Cyclone Idai in Zimbabwe.

The service sector (education, health and WASH) was severely damaged or destroyed by TCI. The total estimated loss for the service sector was US$44.6 m. As shown in Figure 2, about 51% of this amount was attributed to the WASH sector, while the health and education sectors took 34% and 15%, respectively. This shows that the WASH sector needed prioritisation in order to restore or improve its precyclone conditions.

The WASH infrastructure that was severely damaged by TCI were in individual households, health and education facilities and government buildings. The damages included 5830 household toilets, 530 squat hole toilets from 198 schools, 75 submerged wells, 152 boreholes and 17 pumping stations, which were destroyed. Raw sewerage flooded some homes, raising concerns about the health of the local populace. Although the data sources showed statistics for the WASH sector which were separated from the housing, health and education sectors, chances of double counting were highly probable. This is because the WASH infrastructure is closely linked to housing, education centres and health institutions. Therefore, the costs of the WASH sector should be read with caution.

The TCI also damaged nine dams and silted two irrigation canals. Table 3 shows the aggregate of WASH facilities that were either destroyed or damaged by the cyclone.

**TABLE 3:** WASH facilities damaged or destroyed by Tropical Cyclone Idai in Zimbabwe.

WASH facility	Number of facilities destroyed or damaged
Boreholes	152
Piped water schemes	476
Springs	1330
Deep wells	75
BVIP: latrines	7394
Water closet	32

WASH, water, sanitation and hygiene; BVIP, ventilated improved pit latrine.

Within the education sector, classroom blocks and staff houses belonging to 26 schools were destroyed, while 44 others had their roofs either damaged or blown off. The total estimate for restoring the damaged or destroyed education infrastructure was US$6.6 m. However, the actual loss could be less than this figure because the costs of the damages to staff housing, and the related WASH could have been captured under the housing and WASH sectors. This problem is likely to be encountered where the staff quarters are situated in residential areas, not at the schools. Some teaching and learning materials worth US$3 381 565 were also damaged or washed away. As a result, these schools were forced to close a month before the end of the first term.

A total of 182 health institutions (hospitals and rural health centres) were either damaged or destroyed by TCI. The medical equipment, tools, consumables and drugs were also destroyed. The total cost for rebuilding the affected heath infrastructure and restocking the lost equipment and consumables was estimated at US$15 m. However, determining the actual cost of the medical equipment, tools, consumables and drugs which existed before the disaster was very difficult. The tendency of over-reporting cannot be ruled out. Therefore, the estimated loss for the health sector could have been more or less than US$15 m. Eighty per cent of the affected health institutions were in Chimanimani district.

### Direct housing loss

About US$205.3 m loss was induced by TCI in the housing sector, where an estimated 17 715 rural and urban housing units were either destroyed or damaged. The housing units included scattered traditional dwellings made of flammable materials such as poles and grass and mixed, detached or semi-detached and flats or town houses, which were built of more modern materials. Of these housing units, 6795 were in Chimanimani, 6579 in Chipinge and the remainder in other districts of the country. The combined forces of rock-falls, uprooted trees falling on buildings and a windstorm that blew off the roofs were the major causes of damage. One notable residential area that was totally destroyed is the Ngangu township of Chimanimani district, where 500 houses were buried by mudslides. Some of the houses were poorly reinforced or not reinforced at all, a situation which led to the structural damages. However, the value of the property in the damaged housing units could not be ascertained from the surviving members of the affected families.

As observed in the service sector, the loss in the housing sector probably includes some loss aspects of the WASH and education sectors. This is because the construction of housing units would be incomplete without the WASH infrastructure. In addition, some of the education infrastructures that were destroyed or damaged by TCI were situated in close proximity to or in townships, making it a challenge to separate them. Therefore, the estimated loss of the housing sector could be less than the US$205.3 m.

### Direct critical infrastructure loss

**FIGURE 3:**
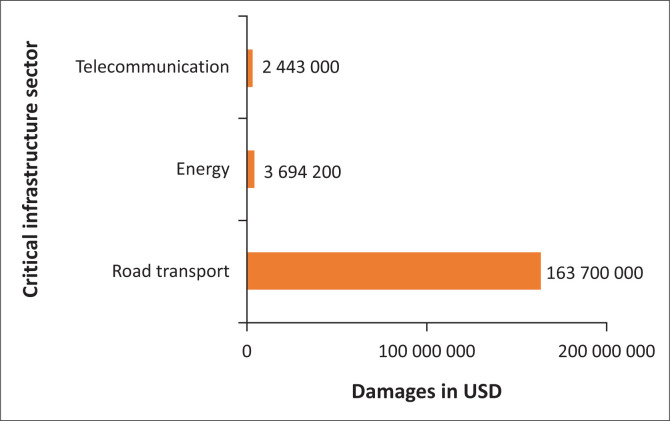
Direct losses in critical infrastructure attributed to Tropical Cyclone Idai in Zimbabwe.

The TCI induced about US$169 837 000 of losses in critical infrastructure (Figure 3). The transport sector was the hardest hit, where about 95% of the road network and 10 bridges were badly damaged or destroyed. About 865 km of roads were either damaged or destroyed. Some of the damages included the eroded road sections, shoulders, lanes and pavement materials; clogged culverts, drifts and inverts; and road sections blocked by mudslides, rockfalls and landslides. As a result, economic activities were disrupted, while the search and rescue operations and disaster relief supplies were delayed. For example, the Ngangu township was cut off for some weeks because of four bridges which were washed away. Most of the bridges that were washed away were those constructed after the country’s independence in 1980, a situation which shows poor building and design practices.

Some of the roads connecting villages and townships in Chimanimani and Chipinge districts were unpaved, a situation that explains why the road damage was very great. In such situations, using the replacement costs is not ideal for the road infrastructure because that would simply return unpaved roads to their predisaster status, which succumbed to the TCI. Probably what is needed is to apply the principle of ‘building back better’, where the road infrastructure is paved and made resistant to the devastating effects of floods. However, doing so would come with increased costs. Consequently, the costs of replacing the road infrastructure would surpass the current estimate of about US$170 m.

The electricity transmission and distribution infrastructure were severely damaged in Chimanimani, Chipinge, Mutare and Rusape districts. The damage involved 33.6 km of MV network, 88.9 km of 33 kV network, 106.2 km of 11 kV network and 40 secondary substations. The estimated cost of the damages was about US$3.7 m (Table 2). The loss of power impacted services to households, businesses, public health and education institutions, communication boosters, the sewer system, water pumping stations and irrigation schemes. Likewise, the telecommunication sector was also damaged to an estimated cost of about US$2.4 m. The cost included repairs and replacement of poles and cables that were damaged or washed away, the damaged parts of mobile telephone boosters and the rehabilitation of communication equipment, including installation of radio and TV antennas. However, the cost of network disruptions because of loss of electricity could not be established.

### Direct cultural heritage loss

An estimated US$800 000 loss was inflicted on cultural heritage sites. The loss included rehabilitating 108 buildings and monuments in the most affected districts and their movable elements, as well as nine sacred sites (pools and springs).

### Total direct economic losses

The total estimate for the direct economic losses attributed to TCI amounted to about US$600 m (Table 4). As shown in Table 4, about 36% of the estimated losses went into the housing sector, while the critical infrastructure nearly consumed 30%. The total direct economic loss in relation to the GDP of the country in 2019 was estimated at 0.03%. Although this figure may look very small, the impact of the TCI on the districts of Chimanimani and Chipinge was huge. The two districts were really ravaged by TCI.

**TABLE 4:** Total direct economic loss attributed to Tropical Cyclone Idai in Zimbabwe.

Economic sector	Estimated damage (USD)	% Contribution
Agriculture	155 392 960	26.98
Services (education, health, WASH)	44 600 000	7.74
Housing	205 300 000	35.65
Critical infrastructure	169 837 000	29.49
Cultural heritage	800 000	0.14
**Grand total**	**575 929 960**	**100**
Direct economic loss in relation to GDP ($19.28 bn)	0.03	-

GDP, gross domestic product.

## Discussion

This study explicitly adapted the OIEWG methodology for Target C of the SFDRR in order to assess the direct economic loss induced by TCI in Zimbabwe. The methodology used a sectoral approach, which included the agriculture sector, services (education, health and WASH), housing, critical infrastructure (energy, road transport and telecommunication) and cultural heritage. The findings revealed that TCI inflicted huge losses in most sectors of the economy. Nearly two-thirds of the losses were channelled through the housing and critical infrastructure, while the agriculture sector lost more than a quarter of the total estimated direct economic losses. As with many economic impact analyses of disasters (Merz et al. 2010; Okuyama 2007), the accuracy of the current estimates can be debated depending on the methodology used and availability of data. For example, the value of the assets in the damaged housing units and other buildings could not be established because of the unavailability of such data across the affected sectors. However, the sectoral analysis approach showed that Zimbabwe needs to prioritise housing, critical infrastructure and agriculture during the recovery and reconstruction phases from TCI. As Zimbabwe’s economy is strongly based on farming, which accounts for approximately 12% of the total GDP (Frischen et al. 2020), the restoration of agricultural livelihoods would contribute to the attainment of the sustainable development goals (SDGs) in Zimbabwe, especially the first three SDGs related to eliminating poverty and hunger and promoting good health and well-being. The sectoral analysis approach also underlined the need to strengthen future mitigation efforts in the housing, agriculture and critical infrastructure sectors, amongst others.

The huge economic losses from TCI were a function of several factors, including the physical characteristics of the cyclone itself, its temporal scale, economic activities of the two most affected districts of Chimanimani and Chipinge and their topographies. As a high-magnitude storm (Category IV storm according to the Saffir-Simpson scale), TCI generated strong surface winds and heavy rains that caused mudslides and rock falls, which damaged housing units, crops, road infrastructure and service sectors, amongst others (Mavhura 2020). The cyclone made its landfall just prior to the harvesting of summer crops, thereby destroying many crops such as maize and bananas. During that time, most soils were already saturated with water, conducive conditions for mudslides and rockfalls which destroyed the built environment (Chanza et al. 2020). River discharge was also high, to the extent that floods swept away roads, bridges and fields. Most of the damages occurred in densely populated townships located in mountainous places with enhanced economic activities, as well as in farms where crops were about to be harvested. In view of the high likelihood of future climate and weather-related disasters in Southern Africa (Chikoore, Vermeulen, & Jury 2015), Zimbabwe faces the risk of economic losses if it fails to adopt and effectively implement adequate DRR strategies. Strengthening DRR strategies in the built environment, including improving the building standards and retrofitting buildings using the ‘build back better’ (3Bs) principle, has been found to be critical in Southern Africa Development Community countries including Zimbabwe (Dube et al. 2021; Owusu-Sekyere, Lunga & Karuaihe 2021). Like Australia, Nepal and Sri Lanka, Zimbabwe can use the 3Bs principle as an aid in determining the post-TCI recovery, reconstruction and rehabilitation best practices (Fernandez & Ahmed 2019). Also critical is the need to strengthen cyclone forecasting and warning systems as a DRR strategy. Zimbabwe can learn from countries such as Hong Kong that have made advances in cyclone forecasting and warning systems and constructing cyclone-resilient infrastructure in order to minimise direct damages (Wong & Choy 2018).

The study used the replacement costs in order to estimate the direct economic losses. This involved calculating the cost of repairing or replacing the damaged or destroyed goods with materials similar to the original ones used before the cyclonic disaster (Natho & Thieken 2018). Such replacement costs were calculated using the OIEWG methodology developed for monitoring progress in achieving the SFDRR Target C (UNDRR 2017). In this study, the methodology worked well because it distinguished between direct and indirect losses induced by TCI. The methodology also worked well with a sectoral analysis approach where uniform indicators of damage and destruction of tangible assets were used. However, the OIEWG methodology was linear in nature and failed to factor in changes in prices of goods and services soon after the disaster. Therefore, the actual replacement estimates might have been higher than what has been provided in this study. Future studies will have to use other methods that factor in price changes from the local markets. The OIEWG methodology also provided room for double counting. For example, losses in the WASH sector were estimated independently from the housing and other services sectors, yet the WASH infrastructure is closely attached to such sectors.

As a result of limited data availability (quantity and quality), this study only calculated the direct economic losses induced by TCI. In some cases, most of the value of the assets in damaged or destroyed houses and building was not included because of insufficient data. Yet the true costs of disasters also include the value of assets in the damaged sectors and the indirect losses which arise from interruptions of the supply chain induced by the disaster (Okuyama 2007). Although calculating the value of the damaged assets places an additional burden on data collection, and indirect losses are difficult to measure, the two are critical in informing DRR policy and practice (Merz et al. 2010). This is because some disasters create nonlinear losses in the demand and supply sides of industry and commerce (Xie et al. 2014a). Hence, data collection needs to be rigorous and future studies that focus on sectoral indirect losses induced by disasters are needed in order to better inform DRR policy and practice.

## Conclusion

This study adapted the OIEWG methodology for Target C to determine the direct economic losses induced by TCI in Zimbabwe. The methodology used a sectoral analysis approach. The results revealed that TCI inflicted huge losses in most sectors of the economy, notably the housing, agriculture and critical infrastructure including road transport, energy and telecommunication. It can be concluded that the OIEWG methodology works well in estimating direct economic losses attributed to disasters. The sectoral extent of damages induced by TCI indicated that Zimbabwe needs to prioritise the housing, critical infrastructure and agriculture during the recovery and reconstruction phases. The same approach also underlined the need to strengthen future mitigation efforts in the housing, agriculture and critical infrastructure sectors, amongst others. There is a need to adopt and effectively implement adequate DRR strategies in the built environment. Strengthening DRR strategies is critical, including improving the building standards using the 3Bs principle. Also critical is the need to strengthen cyclone forecasting and warning systems and construct cyclone-resilient infrastructure in order to minimise direct damages.
